# Validation of ‘Somnivore’, a Machine Learning Algorithm for Automated Scoring and Analysis of Polysomnography Data

**DOI:** 10.3389/fnins.2019.00207

**Published:** 2019-03-18

**Authors:** Giancarlo Allocca, Sherie Ma, Davide Martelli, Matteo Cerri, Flavia Del Vecchio, Stefano Bastianini, Giovanna Zoccoli, Roberto Amici, Stephen R. Morairty, Anne E. Aulsebrook, Shaun Blackburn, John A. Lesku, Niels C. Rattenborg, Alexei L. Vyssotski, Emma Wams, Kate Porcheret, Katharina Wulff, Russell Foster, Julia K. M. Chan, Christian L. Nicholas, Dean R. Freestone, Leigh A. Johnston, Andrew L. Gundlach

**Affiliations:** ^1^The Florey Institute of Neuroscience and Mental Health, Parkville, VIC, Australia; ^2^Somnivore Pty. Ltd., Parkville, VIC, Australia; ^3^Laboratory of Autonomic and Behavioral Physiology, Department of Biomedical and Neuromotor Sciences, University of Bologna, Bologna, Italy; ^4^PRISM Laboratory, Department of Biomedical and Neuromotor Sciences, University of Bologna, Bologna, Italy; ^5^Center for Neuroscience, Biosciences Division, SRI International, Menlo Park, CA, United States; ^6^School of BioSciences, The University of Melbourne, Parkville, VIC, Australia; ^7^School of Life Sciences, La Trobe University, Bundoora, VIC, Australia; ^8^Avian Sleep Group, Max Planck Institute for Ornithology, Seewiesen, Germany; ^9^Institute of Neuroinformatics, University of Zurich and ETH Zurich, Zurich, Switzerland; ^10^The Sleep and Circadian Neuroscience Institute (SCNi), Nuffield Department of Clinical Neurosciences, University of Oxford, Oxford, United Kingdom; ^11^Melbourne School of Psychological Sciences, The University of Melbourne, Parkville, VIC, Australia; ^12^Institute of Breathing and Sleep, Austin Health, Heidelberg, VIC, Australia; ^13^Department of Medicine, St. Vincent’s Hospital, The University of Melbourne, Fitzroy, VIC, Australia; ^14^Biomedical Engineering, The University of Melbourne, Parkville, VIC, Australia; ^15^Florey Department of Neuroscience and Mental Health, The University of Melbourne, Parkville, VIC, Australia

**Keywords:** machine learning algorithms, polysomnography, signal processing algorithms, sleep stage classification, wake–sleep stage scoring

## Abstract

Manual scoring of polysomnography data is labor-intensive and time-consuming, and most existing software does not account for subjective differences and user variability. Therefore, we evaluated a supervised machine learning algorithm, Somnivore^TM^, for automated wake–sleep stage classification. We designed an algorithm that extracts features from various input channels, following a brief session of manual scoring, and provides automated wake-sleep stage classification for each recording. For algorithm validation, polysomnography data was obtained from independent laboratories, and include normal, cognitively-impaired, and alcohol-treated human subjects (total *n* = 52), narcoleptic mice and drug-treated rats (total *n* = 56), and pigeons (*n* = 5). Training and testing sets for validation were previously scored manually by 1–2 trained sleep technologists from each laboratory. *F*-measure was used to assess precision and sensitivity for statistical analysis of classifier output and human scorer agreement. The algorithm gave high concordance with manual visual scoring across all human data (wake 0.91 ± 0.01; N1 0.57 ± 0.01; N2 0.81 ± 0.01; N3 0.86 ± 0.01; REM 0.87 ± 0.01), which was comparable to manual inter-scorer agreement on all stages. Similarly, high concordance was observed across all rodent (wake 0.95 ± 0.01; NREM 0.94 ± 0.01; REM 0.91 ± 0.01) and pigeon (wake 0.96 ± 0.006; NREM 0.97 ± 0.01; REM 0.86 ± 0.02) data. Effects of classifier learning from single signal inputs, simple stage reclassification, automated removal of transition epochs, and training set size were also examined. In summary, we have developed a polysomnography analysis program for automated sleep-stage classification of data from diverse species. Somnivore enables flexible, accurate, and high-throughput analysis of experimental and clinical sleep studies.

## Introduction

Sleep is one of the most critical physiological processes for all species with a nervous system, ranging from jellyfish and flatworms (Lesku and Ly, 2017; Omond et al., 2017) to complex mammals. In humans, sleep is essential for optimal cognitive performance, physiological processes, emotional regulation, and quality of life, and research consistently demonstrates that biological factors leading to disrupted sleep have dramatic effects on health and well-being (Spiegel et al., 2005; Sabanayagam and Shankar, 2010; Watanabe et al., 2010). However, basic and clinical research on sleep has lagged, as the complex dynamics of sleep make it difficult to study. The characterization of sleep states is cardinal to the survey of both physiology and therapeutics; however, the classification of sleep into component stages (or sub-states) has been a challenge since the inception of sleep research (Rechtschaffen and Kales, 1968). Currently, sleep stage scoring remains generally slow, laborious and tedious, and it can be highly subjective. It is therefore the primary bottleneck preventing the sleep research field from flourishing, particularly in light of rapid improvements in polysomnography-related hardware and the emergence of Big Data.

Human sleep stage scoring criteria are currently standardized, following the latest update from the American Academy of Sleep Medicine (AASM) (Berry et al., 2012, 2017). Despite this, scientists trained through the AASM Inter-scorer Reliability Program, exhibited an overall inter-scorer agreement of 82.6%, which decreases to 72–60% for N1, N3 (Rosenberg and Van Hout, 2013) or sleep spindles alone (Wendt et al., 2015). Recent findings suggest that inter-scorer variability (of human polysomnography data) was largely attributed to scoring differences stemming from a large number of equivocal epochs that could legitimately be assigned to multiple sleep stages (Younes et al., 2016). This is a major concern, since discrepancies and inter-scorer disagreements would multiply with experimental parallelization. While this view pertains to human sleep stage scoring, animal sleep scoring is even more problematic because there are no standards comparable to the 10–40 system for electrode placement in humans or an inter-scorer agreement program available. These factors limit the reproducibility of sleep studies, affect overall throughput and underscore the need to develop automated scoring solutions. Animal sleep research has lacked an established platform for automated sleep scoring for multiple reasons, including poor generalization accuracy compared to manual scoring, low user-friendliness for non-engineer users, and discordances within the field due to the subjective nature of sleep scoring. Furthermore, animal sleep-wake states are generally scored as only wake, rapid eye movement (REM) non-REM (NREM), and stages within NREM are not further delineated as they are in humans. Moreover, the majority of automated solutions have only been validated on baseline recordings and frequently for only one species, with some notable exceptions that validated data from both rats and mice (Rytkonen et al., 2011; Bastianini et al., 2014). To date, no analysis method has been validated across recordings from multiple and distantly related species, such as mouse, rat, bird, and human.

Automated analysis protocols have been attempted extensively throughout the last five decades and have been validated using human (Svetnik et al., 2007; Sinha, 2008; Anderer et al., 2010; Nigro et al., 2011; Penzel et al., 2011; Khalighi et al., 2012; Malhotra et al., 2013; Stepnowsky et al., 2013; Kaplan et al., 2014; Koupparis et al., 2014; Punjabi et al., 2015; Wang et al., 2015; Hassan and Bhuiyan, 2017; Koolen et al., 2017; Sun et al., 2017) or rodent data (Crisler et al., 2008; Gross et al., 2009; Stephenson et al., 2009; Rytkonen et al., 2011; McShane et al., 2013; Sunagawa et al., 2013; Bastianini et al., 2014; Kreuzer et al., 2015; Rempe et al., 2015; Gao et al., 2016), with some success, but generally limited adoption by the field. The reasons for this mixed profile are numerous and include: rigidity in the classification, which is unable to accommodate individual differences in polysomnographic data; inadequate ‘user-friendliness’ for users not proficient in software engineering; and inadequate validation, rarely using ‘non-control’ subjects, and not analyzing biological end-measures, limiting metrics to those solely used by computational engineers or statisticians.

Therefore, to address this long-standing need, we developed a supervised machine learning algorithm, Somnivore, which extracts features from a variety of physiological input channels following a brief session of manual scoring, to provide flexible, accurate, and high-throughput analysis of diverse polysomnography data to accelerate the visual wake-sleep scoring process. Validation of the precision and sensitivity of the algorithm generalization (i.e., Somnivore’s ability to predict sleep stage from a learned model built on training sets) were examined in relation to manual scoring by a trained and experienced sleep researcher from each laboratory. For one of the human datasets, we also examined performance in relation to manual inter-scorer agreement of two sleep researchers. Importantly, diverse polysomnography data were used, including data from young, aged, and mildly, cognitively impaired humans; genetically modified (narcoleptic) and drug-treated rodents; and pigeons, in addition to baseline control data.

Specifically, the aims were to: (i) compare generalization of automated scoring versus manual scoring (and versus inter-scorer agreement for one human dataset); (ii) assess the impact of transition epochs on generalization; (iii) examine generalization when learning is performed using single channels; and (iv) assess the impact of different training set sizes on generalization across pooled data in each species.

## Materials and Methods

In order to introduce the relevant methodological details associated with the aims of the study, we first describe the methods used for *in vivo* data collection in humans, mice, rats and pigeons and their manual scoring, followed by the software architecture of Somnivore, the supervised training phase and tests of algorithm generalization versus manual scoring, and the statistical analyses for validating algorithm generalization. All data are represented as mean ± SEM, unless otherwise stated.

### *In vivo* Polysomnography Data Collection

#### University of Melbourne Human (UMH) Cohort Data

All experiments were conducted with prior approval of The University of Melbourne Human Subjects Ethics Committee and informed consent was obtained prior to the screening interview. The UMH cohort data was obtained from mixed male and female participants (*n* = 12), aged between 18 and 21 years, and were extracted from published studies (Chan et al., 2013, 2015). Briefly, after assessing drug use, socioeconomic status, and family history of alcoholism, subjects were introduced to a single blind, repeated measures design with two levels: placebo and alcohol administration. After one adaptation night for habituation, subjects attended two experimental non-consecutive nights at the Melbourne School of Psychological Sciences Sleep Laboratory, The University of Melbourne, Australia. One experimental night involved the administration of a pre-sleep dose of alcohol (vodka mixed with orange juice) designed to achieve a 0.1% peak breath alcohol concentration. The other night involved the administration of a placebo (orange juice with a straw dipped in vodka). The concentration of vodka in the alcoholic drink was determined according to weight, height and total body water measurement. Female participants were tested in the course of their menstrual mid-follicular phase, and those on a contraceptive pill were tested during their scheduled week on placebo pill. Six EEG electrodes (F3:A2, F4:A1, C3:A2, C4:A1, O1:A2, O2:A1), left and right electrooculogram (EOG), submental electromyogram (EMG), and electrocardiogram (ECG) were recorded. EEG leads were recorded to a single, midline forehead reference and then digitally re-referenced to the contralateral ear. All channels were captured at a 512 Hz sampling frequency and displayed with a 0.3–30 Hz band-pass filter. Data were collected using Compumedics hardware (Siesta) and software (Profusion PSG3) (Compumedics Ltd., Abbotsford, VIC, Australia), and manually scored in 30 s epochs according to AASM criteria, by two experienced scorers from the Sleep Laboratory at the University of Melbourne, both blinded to the experimental conditions. Recording durations ranged from 7 – 12 h (mean 10.04 ± 0.30 h; *n* = 24 recordings from 12 participants for 2 nights).

#### University of Oxford Human (UOH) Cohort Data

The UOH cohort represents data compiled by collecting baseline recordings from a variety of male and female subjects, including healthy young adults (HYA, *n* = 12, 20–34 year-old) extracted from an unpublished study (Porcheret et al., 2019), as well as healthy older adults (HOA, *n* = 9, 65–78 year-old) and older adults diagnosed with mild cognitive impairment using established criteria (MOA, *n* = 7, 72-84 year old), extracted from a published study (Wams et al., 2017). All recordings were collected ambulatory. The HYA study was conducted in accordance with the Declaration of Helsinki and approved by the National Research Ethics Service (NRES) committee, East of England, Hatfield (REC number 14/EE/0186). The HOA and MOA study was conducted in accordance with the Declaration of Helsinki and approved by the NRES committee, South East England, Berkshire (REC number 09/H0505/28). A summary of the cohort composition is provided in Supplementary Table 1.

Briefly, polysomnographic recordings were made using the Actiwave system (CamNtech Ltd, Cambridge, United Kingdom) and a nine electrode montage: Fz:A2; Cz:A2; Pz:A2; Oz:A2 (recorded using an EEG/ECG 4 unit), Cz:A1 (recorded using an EEG/ECG 1 unit), EOG1:A2; EOG2:A1 (recorded using an EEG/ECG 2 unit) and EMG1:chin; EMG2:chin (recorded using an EMG 2 unit). The EEG and EOG channels had a sampling rate of 128 Hz and EMG channels had a sampling rate of 256 Hz. Recording durations ranged from 11 to 14 h (mean 11.75 ± 0.17 h; *n* = 28 recordings). Data were manually scored in 30 s epochs by an experienced scorer at the University of Oxford, according to AASM criteria (Wams et al., 2017).

#### University of Bologna Mouse (UBM) Cohort Data

Experiments were conducted with prior approval of the Ethical-Scientific Committee of the University of Bologna, in accordance with the European Union Directive (86/609/EEC) and under the supervision of the Central Veterinary Service of the University of Bologna and the National Health Authority. A summary of all subjects in the rodent data validation studies is provided in Supplementary Table 2.

The UBM cohort represents data of two age-matched sub-cohorts extracted from a published study (Bastianini et al., 2011). The data were from adult hemizygous hypocretin-ataxin3 transgenic mice (*n* = 9), with a C57BL/6J genetic background, that exhibited the selective postnatal ablation of hypocretinergic neurons and wild type C57BL/6J littermates (*n* = 9) with intact hypocretinergic neurons (Bastianini et al., 2011). Transgenic mice expressed the neurotoxin ataxin-3 under the control of the hypocretin promoter, thus, this toxin compound only accumulated in hypocretinergic neurons leading to their complete ablation before adulthood. This procedure is designed to mirror the pathogenesis of narcolepsy in human patients. Surgery and recordings were performed as described (Hara et al., 2001).

Mice were implanted with two pairs of electrodes to acquire EEG and EMG signals. In particular, 2 stainless-steel screws (2.4 mm length, Plastics One, Roanoke, VA, United States) were positioned in contact with the dura mater through burr holes in the frontal (2 mm anterior and 2 mm lateral to bregma) and parietal (2 mm anterior and 2 mm lateral to lambda) bones to obtain a differential EEG signal. A second pair of Teflon-coated stainless-steel electrodes (Cooner Wire, Chatsworth, CA, United States) was inserted bilaterally in the nuchal muscles to obtain a differential EMG signal. All the electrodes were connected to a miniature custom-built socket, which was cemented to the skull with dental cement (Rely X ARC, 3M ESPE, Segrate, Milano, Italy), and dental acrylic (Respal NF, SPD, Mulazzano, Italy). After at least 10 days of recovery, mice were tethered by cable on a rotating swivel and a balanced weight to allow for free-movement and let to habituate to the recording setup for 1 more week. Both EEG and EMG were respectively filtered at 0.3–100 Hz and 100–1000 Hz and captured at a 128 Hz sampling rate. Recordings were continuously collected for 3 days and manually scored in 4 s epochs using visual scoring criteria by an experienced scorer at the University of Bologna (Silvani et al., 2009). The final 30 h of each scored recording was used for these validation studies. Analysis of sleep microstructure was performed with a threshold of 12 s (i.e., three consecutive 4-s epochs) with no other contextual scoring rules (e.g., REM can be scored consecutive to wake). Epochs containing artifacts or undetermined states in manually scored hypnograms were excluded from the automated scoring.

### University of Bologna Rat (UBR) Cohort Data

Experiments were conducted with prior approval of the Ethical-Scientific Committee of the University of Bologna, in accordance with the European Union Directive (86/609/EEC) and under the supervision of the Central Veterinary Service of the University of Bologna and the National Health Authority.

The UBR cohort represents data from two age-matched sub-cohorts extracted from a published study (Supplementary Table 2) (Cerri et al., 2013). Data were from male Sprague-Dawley rats microinjected in the rostral ventromedial medulla (RVMM) with the GABA_A_ agonist, muscimol in vehicle solution (*n* = 8) or vehicle alone (*n* = 8) (Cerri et al., 2013). Briefly, rats were implanted with electrodes for EEG (first electrode: 3.0 mm anterior - 3.0 mm lateral; second electrode: 4.0 mm posterior – 1.5 mm lateral to bregma) and nuchal EMG; a thermistor mounted in a stainless steel needle to record deep brain temperature (T_brain_); and a microinjection cannula positioned stereotaxically to deliver vehicle or muscimol into the RVMM. EEG was captured at a 1 kHz sampling rate and filtered at 0.3 Hz highpass and 30 Hz lowpass; EMG was captured at a 1 kHz sampling rate and filtered 100 Hz highpass and 1 kHz lowpass; T_brain_ was captured at 100 Hz sampling rate and filtered 0.5 Hz highpass. For treatment, each rat received 6 injections, one per hour, of either muscimol (MT group, 1 mM muscimol in 100 nl saline solution (0.9% NaCl w/v) or saline alone (100 nl). EEG, EMG and T_brain_ were captured for a total of 24 h, and manually scored at 1 s resolution, taking into account the EEG power spectrum (calculated from a 4 s long, 1 s sliding window) by an experienced scorer at the University of Bologna, using published criteria (Cerri et al., 2005). Scoring consistency rules were set at 4 s for wake and 8 s for NREM/REM sleep, while no other contextual scoring rules were applied (Cerri et al., 2013). All UBR recordings were 24 h duration.

#### SRI International Rat (SRI) Cohort Data

Experimental procedures involving animals were approved by SRI International’s Institutional Animal Care and Use Committee and were in accordance with National Institutes of Health guidelines. The SRI cohort represents data from 3 sub-cohorts extracted from one published study (Morairty et al., 2014) and one unpublished study (Supplementary Table 2). Data were from male Sprague-Dawley rats that received an intraperitoneal injection of vehicle or caffeine (10 mg/kg) at circadian time 0 (CT0) during lights on (SRI-CAF; *n* = 7), vehicle or zolpidem (30 mg/kg) at CT12 during lights off (SRI-ZOL; *n* = 2), and vehicle or almorexant (100 mg/kg) at CT12 during lights off (SRI-ALM; *n* = 2) (Morairty et al., 2014). Therefore, a total of 22 scored recordings were used in these validation studies.

Briefly, rats were implanted intraperitoneally with a sterile telemetry transmitter (DSI F40-EET, Data Sciences Inc., St Paul, MN, United States). Biopotential leads were guided subcutaneously to the head (for EEG recording) and neck (for EMG recording). The 2 EEG electrodes were placed in reference to bregma at 1.5 mm AP, 1.5 mm ML and at -4.0 mm AP, 3.0 mm ML. Recordings were made for 12 h in the zolpidem and almorexant cohorts and 6 h in the caffeine cohort. Data were collected in 10 s epochs and scored manually by an experienced scorer at SRI International, using DSI NeuroScore software (Data Sciences Inc.). There were no restrictions on what an epoch could be scored, regardless of what the previous epoch was scored. All treatments were administered orally; vehicle was 1.25% hydroxypropyl methylcellulose, 0.1% dioctyl sodium sulfosuccinate and 0.25% methylcellulose in water. All SRI recordings were 6–12 h duration.

#### Max Planck Institute Pigeon Cohort Data

Experiments were conducted with prior approval by the Government of Upper Bavaria and adhered to the National Institutes of Health standards for using animals in research. This cohort represents data compiled by collecting baseline recordings from Tippler (*Columba livia*) pigeons (*n* = 5), purchased from a local breeder in Upper Bavaria, Germany.

To record the EEG, the pigeons were implanted with electrodes over the anterior (AP +13.0 mm) hyperpallium (L +2.0 mm) and mesopallium (L +6.0 mm) of the left and right hemisphere (Karten and Hodos, 1967). A reference electrode was positioned over the cerebellum, and a ground electrode was implanted over the left hemisphere, halfway between the medial recording electrode and the reference. Birds were given at least 2 weeks to recover after surgery before recordings commenced. EEG signals were obtained using a data logger that records the EEG along with fine/gross head movements measured by a triaxial accelerometer (Neurologger 2A)^1^ (Lesku et al., 2012; Rattenborg et al., 2016). The pigeons were habituated to the data logger prior to the onset of data collection.

Recordings of 42–83 h duration (mean 50.11 ± 8.1 h) were imported into REMLogic (Natus; Embla RemLogic 3.4.0) and were visually scored for wakefulness, NREM and REM using 4 s epochs with the aid of video recordings, by an experienced scorer at La Trobe University. NREM (or slow wave sleep) was scored when more than half of an epoch displayed low-frequency activity with an amplitude approximately twice that of alert wakefulness. In each case, the onset of scored NREM typically corresponded with the onset of sleep behavior (e.g., immobility, head drawn into the chest). REM sleep was characterized by periods of EEG activation (>2 s) occurring in association with bilateral eye closure and behavioral signs of reduced muscle tone (e.g., head dropping, swaying, and sliding of the wings off the side of the body).

### Software Architecture

Somnivore was written in MATLAB (Mathworks, Natick, MA, United States), compiled to run stand-alone and is compatible with Microsoft Windows (version 7, 8, 8.1, and 10; Microsoft Inc., Seattle, WA, United States). It has been designed to classify 4, 10, or 30 s epochs, and the overall analysis procedure is divided into several steps starting with data import, followed by a brief training bout of manual scoring using training sets within each recording, automated wake-sleep stage scoring of the remaining recording using the algorithm-generated learning model, and finally, setting of contextual scoring rules.

All recordings were uploaded to, and analyzed with, a Lenovo ThinkPad laptop W540 with an Intel^®^ Core^TM^ i7-4900MQ central processing unit, 32 GB of total physical memory, Intel^®^ HD Graphics 4600 principal display adapter, NVIDIA-Quadro K1100M secondary display adapter, and a OCZ-VERTEX4 512 GB solid-state drive.

#### Training Phase

All previously manually scored animal and human data files were provided in .edf or .txt format. The size of the training sets was set *a priori* with the aim of optimizing the classifier around this parameter. Different training set sizes for different epoch lengths were set according to arbitrary amounts of manual training, addressing both assumptions of user-friendliness and preliminary tests of training dynamics in a supervised machine learning model (data not shown). The training set size for 4 s epoch length cohorts (mouse [UBM] and rat [UBR] data collected at the University of Bologna; pigeon data scored at La Trobe University) was set at 100 epochs per stage (Wake, NREM, REM), totalling 6.67 min of manual scoring per state. For the 10 s epoch length cohort (rat data collected at SRI International [SRI]), the training set size was set at 50 epochs per stage (Wake, NREM, REM), totalling 8.33 min of manual scoring per state. For 30 s epoch length cohorts (human data collected at the University of Oxford [UOH] and the University of Melbourne [UMH]), the training set size was set at 40 epochs per vigilance stage (Wake, N1, N2, N3, REM), totalling 20 min of manual scoring per state. Training sets were built by random epoch selection from the manually scored hypnograms.

For cohorts where subjects were tested twice with recordings of baseline activity followed by treatment (UMH and SRI), algorithm generalization from two training sets within each subject were assessed: ‘longitudinal-trained’ training sets comprised of a subset of epochs from the baseline and treatment recordings; and ‘baseline only trained’ training sets comprised of a subset of epochs from the baseline recording only.

### Performance Evaluation of the Algorithm

#### Assessing Agreement Between Automated Scoring and Manual Scoring

Following the training phase and optimization of the learning model for each recording, automated wake-sleep stage scoring of the remaining recording was conducted and its agreement with manual scoring was analyzed. The metric used to ensure the most rigorous evaluation of algorithm generalization accuracy versus manual scoring was the *F*-measure (Powers, 2011).

The *F*-measure, also known as *F*-score or *F*_1_-score, combines precision and sensitivity together as one measure (Powers, 2011). It is one of the most stringent metrics available, as both high sensitivity and high precision are required for high *F*-measure. It is calculated as: $F-measure=2\cdot \frac{sensitivity\cdot precision}{sensitivity+precision}$ where $\mathrm{sensitivity}=\frac{TP}{\left(TP+FN\right)}$ and $\mathrm{precision}=\frac{TP}{\left(TP+FP\right)}$. TP, also called hits, represent data points that were correctly classified in a specific class by the algorithm. False positives (FP) are type I errors, where the algorithm flagged a data point as being a member of the class considered, when in fact the manual scoring classified it as being part of another class. False negatives (FN) are type II errors, or misses, where the algorithm labeled a data point as being part of another class from that considered.

In accordance with the literature on the inter-scorer agreement reported for human sleep data (Kelley et al., 1985; Himanen and Hasan, 2000; Norman et al., 2000; Danker-Hopfe et al., 2004), five arbitrary generalization threshold ratings were set throughout Somnivore’s human validation studies for *F*-measure: intra-scorer ≥ 0.9, excellent ≥ 0.85, strong ≥ 0.8, average ≥ 0.7, and inadequate < 0.7.

Since each level of optimization was determined by a comparison between two subject-matched groups, Student’s paired *t*-tests were used to determine statistical significance, with significance set at *p <* 0.05. For analysis involving two Student’s paired *t*-tests, *a priori* alpha adjustment was applied via Bonferroni correction to a significance level of *p <* 0.025.

Effect sizes were also evaluated via Hedges’ $g:g=\frac{M_{1}-M_{2}}{\sqrt{\mathrm{SD}\_\mathrm{pooled}}}$ where M_1_ is the mean of the first group, *M*_2_ is the mean of the second group, and *SD_pooled* is the pooled standard deviation. The use of *g* over the more established Cohen’s *d* relies on the fact that the former is considered a more rigorous and robust metric for smaller sample sizes (Hofmann et al., 2010). Hofmann et al. (2010) also argued that the magnitude for *g*, just like Cohen’s *d*, was interpretable using the same guidelines set by Cohen (1988), i.e., small 0.2 ≤ *g* < 0.5, medium 0.5 ≤ *g* < 0.8, and large g ≥ 0.8. Effect sizes of g < 0.2 were termed ‘miniscule.’

#### Effects of Treatments and Genetic Phenotype on Algorithm Generalization

Because the UMH human cohort data was manually scored by two investigators, inter-scorer agreement between hypnograms manually scored by scorer 1 or 2 (MS1; MS2) was evaluated, and versus the hypnograms automatically scored by Somnivore using training sets derived from either scorer 1 or 2 (AS1; AS2). To assess effects of alcohol treatment on generalization performance, the manually scored hypnograms of only scorer 1 were assessed using both longitudinal- and baseline only training training sets.

For the mouse and rat data, agreement between manual scoring and algorithm generalization was examined in control animals, in addition to the impact of genetic phenotype or pharmacological treatment. Generalization accuracy of UBM and UBR cohorts were compared between wildtype and transgenic (transgenic) cohorts and vehicle-treatment and muscimol-treatment cohorts, respectively. Since the groups were independent, an independent samples *t*-test was used, with *p <* 0.05 considered significantly different. SRI cohorts, on the other hand, were tested twice, and all treatment groups (caffeine, zolpidem and almorexant) were compared between vehicle and treatment recordings, with both longitudinal-trained and baseline only trained training sets. Since the SRI data represents a within-subjects design, a Student’s paired *t*-test was used. While the SRI cohort was tested twice, Bonferroni correction was not used, to increase test stringency.

#### Effects of Transition Epochs on Algorithm Generalization

Generalization for each species of pooled human (*n* = 52), mouse and rat (*n* = 54) and pigeon (*n* = 5) recordings was assessed in conditions where transition epochs were excluded from the analysis, and compared against the standard configuration inclusive of transition epochs. Transition epochs were defined as the first and the last epoch of each sleep stage bout. For their exclusion, generalization was ignored at the level of transition epochs from the automated scoring versus human scoring, such that the transition epochs from the automated hypnogram were excluded, and the corresponding epochs of the manually scored hypnogram were not analyzed.

#### Effects of Single Input Channels on Algorithm Generalization

For the human cohort data, the impact of training Somnivore’s classifiers with one EEG channel or two EOG channels only, was assessed in pooled human recordings (*n* = 52). Generalization was evaluated in three training input channel configurations: all channels (standard configuration): one EEG channel only (Cz, C3, or C4 based on configuration), and two EOG only. While three Student’s *t*-tests were applied when comparing different training channel configurations correcting statistical significance *a priori* would favor the outcome of the validation by containing type II errors, as performance is expected to fall significantly. Therefore, no Bonferroni correction was applied to increase stringency in the evaluation.

The impact of simplified human sleep stages on generalization was also assessed in pooled human recordings (*n* = 52), including the effects of N1 reclassified as either wake or N2. Classifiers were also trained with simplified human sleep stages to resemble those used in rodent studies, where N1 was reclassified as wake, and N2 and N3 were both reclassified as NREM (W1NREM23); or N1, N2 and N3 were reclassified as NREM (NREM123).

For the mouse and rat cohort data, recordings were pooled (*n* = 54) and evaluated in three training input channel configurations: EEG+EMG, EEG only, and EMG only. Comparisons were evaluated within and between configurations. While multiple Student’s *t*-tests were applied (three for EEG+EMG including all epochs, two for EEG+EMG excluding transitions), correcting statistical significance *a priori* would favor the outcome of the validation by containing type II errors. Therefore, no Bonferroni correction was applied to increase stringency in the evaluation.

For the pigeon cohort data, generalization (*n* = 5) was evaluated in three training input channel configurations: EEG+accelerometer (ACC), EEG only, and ACC only. Comparisons were then evaluated within and between configurations. While multiple Student’s *t*-tests were applied, no Bonferroni correction was applied to increase stringency in the evaluation.

#### Effects of Training Set Size on Algorithm Generalization

‘Training set size’ response curves were generated for pooled human (*n* = 52), mouse and rat (*n* = 54), and pigeon (*n* = 5) cohorts using standard configurations inclusive of transition epochs. For the human data, classifiers were trained with a range of 5–80 training epochs per vigilance stage. Accordingly, three training set sizes were chosen for statistical comparison in the following order: (i) the smallest training set size to output strong overall generalization (*F*-measure > 0.8); (ii) the default training set size for epoch lengths of 30 s; and (iii) the maximum training set size deemed to remain user-friendly.

For the rodent data, classifiers were trained with a range of 5–200 training epochs per vigilance state. Accordingly, four training set sizes were chosen for statistical comparison, in the following order: (i) the smallest training set size to output adequate generalization across all cohorts (*F*-measure ≥ 0.9 across all states); (ii) the default training set size for epoch lengths of 10 s; (iii) the default training set size for epoch lengths of 4 s; and (iv) the maximum training set size for the range considered.

For the pigeon data, classifiers were trained with a range of 5-400 training epochs per vigilance state. Accordingly, four training set sizes were chosen for statistical comparison.

## Results

### Validation of the Algorithm With Human Data

#### Manual Versus Algorithmic Inter-Scorer Agreement

**FIGURE 1:**
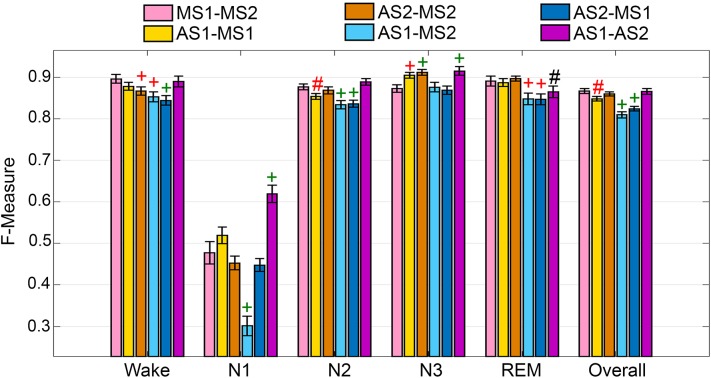
Manual versus automated scoring inter-scorer agreement for UMH cohort. Generalization of all UMH recordings (*n* = 24) between different combinations of manual (MS1, MS2) and automated scoring (AS1, AS2) inter-scorer agreements. Data analyzed by paired *t*-test, represented as mean ± SEM. ^#^*p* < 0.01; ^+^*p* < 0.001. Symbol color represents effect size (g): black, small; red, medium; green, large.

The UMH cohort data was manually scored by two researchers, so agreement between hypnograms manually scored by scorer 1 or 2 (MS1; MS2) versus algorithmic hypnograms automatically scored using training data drawn from manual scoring of scorer 1 or 2 (AS1; AS2) was evaluated. All comparative combinations of manually and automatically scored hypnograms were assessed against the gold-standard, which is the inter-scorer agreement between MS1 and MS2 (MS1-MS2) (Figure 1). Algorithm generalization (i.e., automated wake-sleep stage scoring agreement with manual scoring) was strong to excellent (*F*-measure = 0.84-0.90) across all groups for wake, with only AS2-MS2, AS1-MS2, and AS2-MS1 comparisons significantly lower. N1 generalization across all comparisons was inadequate (0.30–0.61), with only AS1-MS2 significantly lower, and AS1-AS2 significantly higher. N2 generalization across all comparisons was strong to excellent (0.83–0.89), with only AS1-MS1, AS1-MS2, and AS2-MS1 significantly lower. N3 generalization across all comparisons was excellent to intra-trainer level (0.87–0.92). Interestingly, only AS1-MS1, AS1-MS2, and AS1-AS2 comparisons were significantly different, and all were higher than the gold-standard MS1-MS2, thus performing at intra-trainer level. REM generalization across all comparisons was excellent to intra-trainer level (0.85–0.90), with only AS1-MS2, AS2-MS1, and AS1-AS2 significantly lower, the latter with small effect size. Overall generalization across all comparisons was strong to excellent (0.81–0.87), with only AS1-MS1, AS1-MS2, and AS2-MS1 significantly lower.

#### Generalization Across Experimental Groups

**FIGURE 2:**
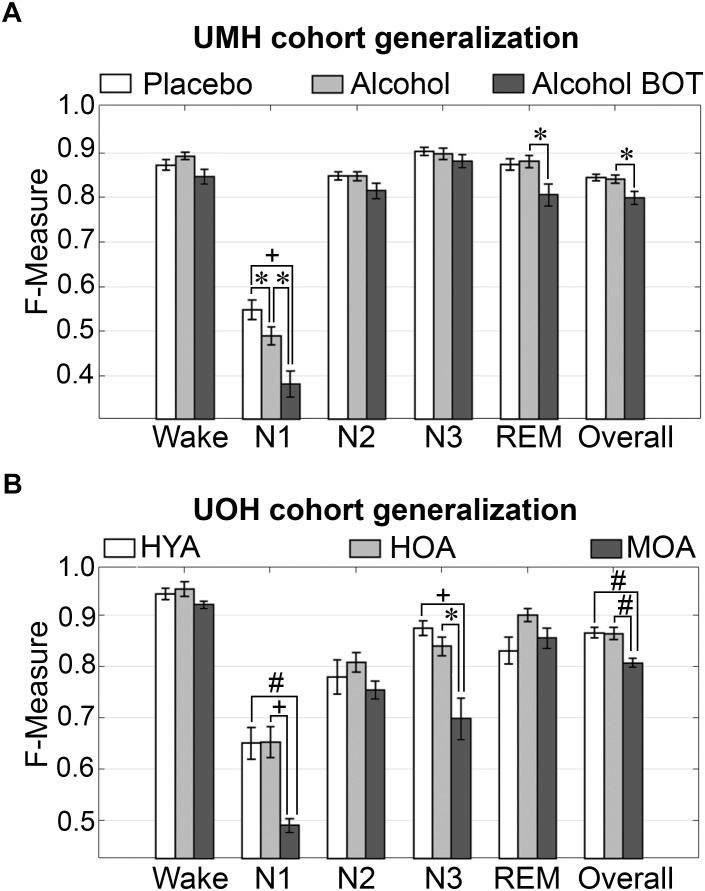
Generalization of UMH and UOH human cohorts across experimental groups. **(A)** Generalization of UMH cohort (*n* = 12) across experimental groups: longitudinal-trained placebo and alcohol, and baseline only-trained alcohol (BOT), analyzed by paired *t*-test. **(B)** Generalization of UOH cohort (HYA, *n* = 12; HOA, *n* = 9; MOA, *n* = 6) across experimental groups, analyzed by independent sample *t*-test. Data represented as mean ± SEM. ^∗^*p* < 0.05; ^#^*p* < 0.01; ^+^*p* < 0.001.

To assess algorithm generalization across UMH experimental groups, the effects of treatment on generalization accuracy, and use of longitudinal and baseline only training sets, the manually scored hypnograms of only scorer 1 were assessed (i.e., AS1-MS1 comparisons). Generalization significantly differed between placebo and alcohol groups only for N1 scoring, which remained inadequate overall and significantly decreased from 0.55 ± 0.02 to 0.49 ± 0.02 (*p* < 0.05) with medium effect size (*g* = 0.78) (Figure 2A). Generalization for the baseline only trained alcohol group (i.e., training sets from placebo recording only) significantly decreased for a number of stages: N1 *F*-measure was inadequate overall and further decreased to 0.38 ± 0.03 compared to longitudinal-trained placebo (*p* < 0.001) or alcohol (*p* < 0.05) groups (i.e., training sets from placebo and alcohol recordings) with large effect size (placebo *g* = 1.8; alcohol *g* = 1.22); REM F-measure significantly decreased only between alcohol longitudinal- and baseline only trained groups, from excellent (0.88 ± 0.01) to strong (0.81 ± 0.02) (*p* < 0.05) with large effect size (*g* = 1.04); while the overall F-measure remained strong between the alcohol longitudinal- and baseline only trained groups, albeit significantly decreased from 0.84 ± 0.01 to 0.80 ± 0.01 (*p* < 0.05) with large effect size (*g* = 0.96).

Generalization did not significantly differ for any individual sleep stage or overall in the UOH cohort between healthy young adults (HYA) and healthy old adults (HOA) groups (Figure 2B). However, generalization for the group of older adults diagnosed with mild cognitive impairment (MOA) decreased for a number of stages: N1 generalization remained inadequate throughout, significantly decreasing from 0.65 ± 0.03 to 0.49 ± 0.01 compared to HYA (*p* < 0.01) and HOA (*p* < 0.001), with large effect sizes (HYA, *g* = 1.69; HOA, *g* = 2.12); N3 F-measure significantly decreased from excellent (HYA, 0.88 ± 0.01) and strong (HOA, 0.84 ± 0.02) to average (MOA, 0.70 ± 0.04) (HYA, *p* < 0.001; HOA, *p* < 0.05) with large effect sizes (HYA, *g* = 2.26; HOA, *g* = 1.65). Overall *F*-measure significantly decreased from excellent (HYA, 0.87 ± 0.01; HOA, 0.86 ± 0.01) to strong (MOA, 0.81 ± 0.0; *p* < 0.01) with large effect sizes (MOA vs. HYA, *g* = 1.86; HOA, *g* = 1.73). Despite some significant decreases in *F*-measure, specifically for N3 (excellent to average) and overall (excellent to strong), the practical relevance of these decreases would be minimal. Thus, treatment or cognitive capacity of human subjects has minimal practical impact on the accuracy of automated wake–sleep stage scoring of this algorithm.

#### Impact of Transition Epochs

**FIGURE 3:**
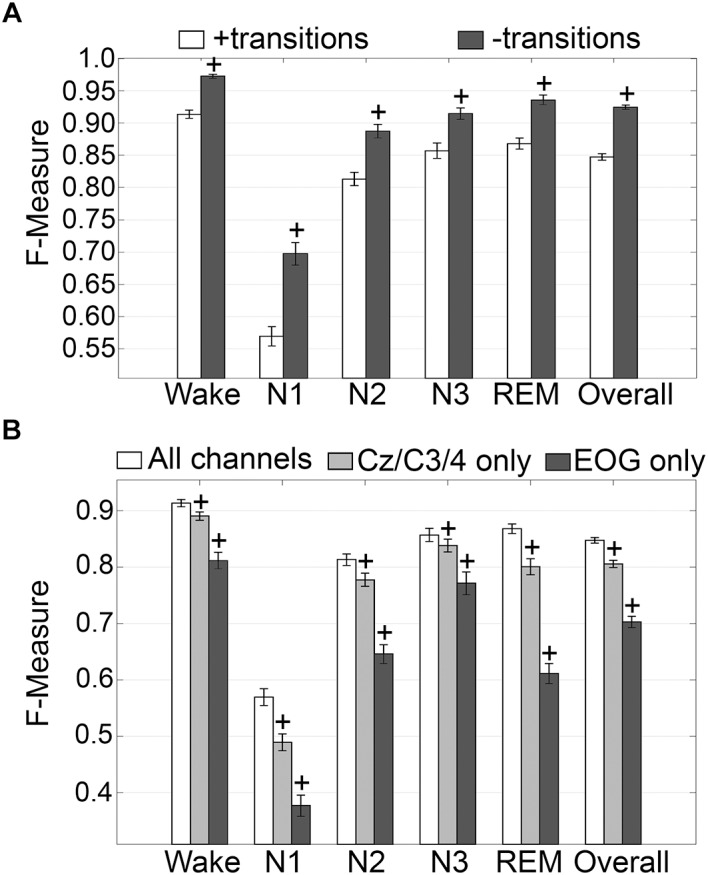
Impact of automated removal of transition epochs, and learning with one EEG channel or two EOG channels on generalization of human recordings. **(A)** Generalization of all human recordings (*n* = 52), with and without consideration of transition epochs. **(B)** Generalization of all human recordings across three different input channel configurations: all channels; Cz/C3/C4 only; or EOG only. Data analyzed by paired *t*-test compared to all channels, and represented as mean ± SEM. +*p* < 0.001.

The impact of transition epochs on generalization across pooled human recordings (*n* = 52) was assessed. Automated removal of transition epochs (i.e., the first and last epoch of each sleep stage bout) resulted in exclusion of 24.6 ± 0.95% of total epochs, which had a significant impact on algorithm generalization (Figure 3A). Generalization of wake remained at intra-scorer level, significantly increased from 0.91 ± 0.01 to 0.97 ± 0.01 (*p* < 0.0001) with large effect size (*g* = 1.63). N1 generalization significantly increased from inadequate (0.57 ± 0.01) to average (0.70 ± 0.02) (*p* < 0.0001) with large effect size (*g* = 1.09). N2 generalization significantly increased from strong (0.81 ± 0.01) to excellent (0.89 ± 0.01) (*p* < 0.0001) with large effect size (*g* = 0.98). N3 generalization significantly increased from excellent (0.86 ± 0.01) to intra-scorer level (0.91 ± 0.01) (*p* < 0.0001) with medium effect size (*g* = 0.75). REM generalization significantly increased from excellent (0.87 ± 0.01) to intra-scorer level (0.94 ± 0.01) (*p* < 0.0001) with large effect size (*g* = 1.14). Overall generalization significantly increased from excellent (0.85 ± 0.01) to intra-scorer level (0.92 ± 0.01) (*p* < 0.0001) with large effect size (*g* = 2.55). These results indicate that the variability of algorithm-generated automated wake-sleep stage scoring largely lies in the determination of transition epochs, particularly in the analysis of N1 stages. In practical terms, automated tagging of transition epochs for subsequent refinement of the machine learning process would potentially provide substantial increases in algorithmic accuracy to intra-scorer level for all stages (except N1) and overall.

#### Generalization on Training With One EEG Channel or Two EOG Channels Only

Generalization using training sets from one EEG channel (Cz, C3, or C4) or two EOG channels only, inclusive of transition epochs, was assessed (Figure 3B). When compared with learning using all available channels, generalization decreased for all stages. Wake generalization significantly decreased from intra-scorer level (0.91 ± 0.01) for all channels configuration to excellent (0.89 ± 0.01) for one EEG channel only (*p* < 0.0001) with small effect size (*g* = 0.46); and strong for EOG channels only (0.81 ± 0.01) (*p* < 0.0001) with large effect size (*g* = 1.24). N1 generalization was inadequate for all configurations, and significantly decreased from 0.57 ± 0.01 to 0.49 ± 0.01 for all channels configuration for one EEG channel only (*p* < 0.0001; *g* = 0.74), and to 0.38 ± 0.02 for EOG channels only (*p* < 0.0001, *g* = 1.57). N2 generalization significantly decreased from strong (0.81 ± 0.01) for all channels configuration to average (0.78 ± 0.01) for one EEG channel only (*p* < 0.0001) with small effect size (*g* = 0.44); and to inadequate for EOG channels only (0.65 ± 0.01) (*p* < 0.0001) with large effect size (*g* = 1.65). N3 generalization significantly decreased from excellent (0.86 ± 0.01) for all channels configuration to strong (0.84 ± 0.01) for one EEG channel only (*p* < 0.001) with small effect size (*g* = 0.22); and to average for EOG channels only (0.77 ± 0.02) (*p* < 0.0001) with medium effect size (*g* = 0.72). REM generalization significantly decreased from excellent (0.87 ± 0.01) for all channels configuration to strong (0.80 ± 0.01) for one EEG channel only (*p* < 0.0001) with medium effect size (*g* = 0.79); and to inadequate for EOG channels only (0.61 ± 0.02, *p* < 0.0001) with large effect size (*g* = 2.56). Overall generalization significantly decreased from excellent (0.85 ± 0.01) for all channels configuration to strong (0.81 ± 0.01) for one EEG channel only (*p* < 0.0001) with large effect size (*g* = 1.02); and average for EOG channels only (0.7 ± 0.01) (*p* < 0.0001) with large effect size (*g* = 2.54). Taken together, these results suggest that a single EEG channel was sufficient to generate strong automated scoring > 0.80 of wake, N3, and REM stages, with scoring N2 marginally lower at 0.78. Furthermore, two EOG channels was sufficient to generate strong automated scoring (>0.80) of wake, and average performance for N3 stages (0.77), but was inadequate for REM. N1 generalization was inadequate overall.

#### Generalization of Sleep Stage Simplified Configurations

**FIGURE 4:**
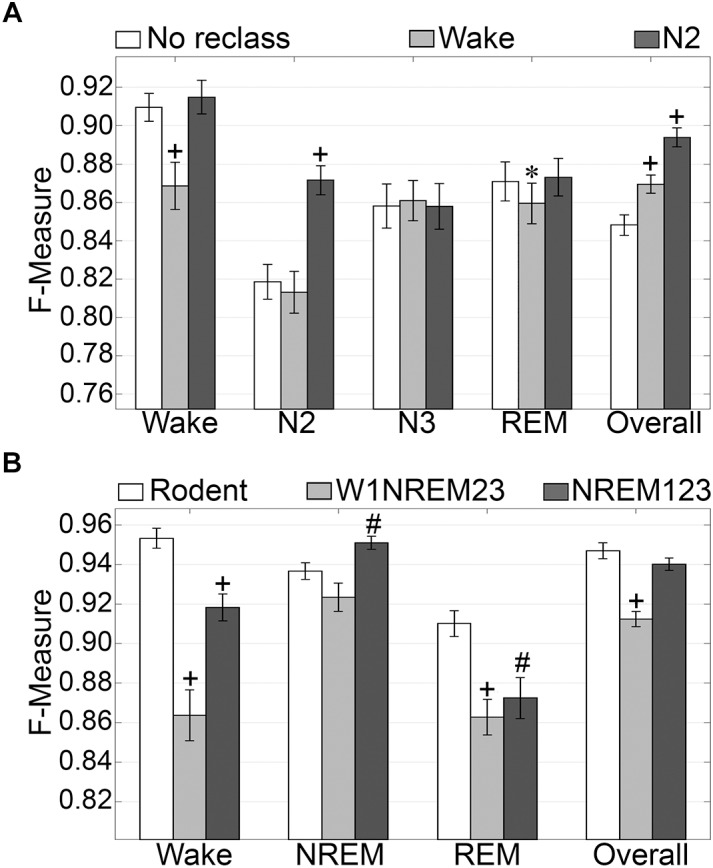
Generalization of human recordings with N1 reclassification or stage simplification versus rodent recordings. **(A)** Comparison of generalization performance of all human recordings (*n* = 52) between standard stage classification and two versions of N1 reclassification: N1 reclassified as wake; N1 reclassified as N2. Data analyzed by paired *t*-test compared to control (no reclassification). **(B)** Generalization of pooled rodent recordings (*n* = 54) versus human recordings (*n* = 52) with two versions of stage simplification: N1 reclassified as wake, N2 and N3 reclassified as NREM (W1NREM23); or N1, N2, and N3 reclassified as NREM (NREM123). Data analyzed by independent samples *t*-test compared to rodent recordings. Data represented as mean ± SEM. ^#^*p* < 0.01; ^+^*p* < 0.001.

N1 is a volatile stage that systematically produces inadequate agreement and is an inherently difficult stage to score (Wendt et al., 2015; Younes et al., 2016). Thus, we assessed the effects of reclassifying N1, to either wake or N2 on generalization accuracy. The NREM stages of rodent data are not delineated into the sub-stages used for humans. Thus, we also assessed the effects of simplifying the human NREM stages to those in rodent models. Reclassifying N1 as N2 did not significantly affect wake generalization, but when N1 was reclassified as wake, generalization significantly decreased from intra-scorer level (0.91 ± 0.01) to excellent (0.86 ± 0.01) (*p* < 0.001) with medium effect size (*g* = 0.56) (Figure 4A). N2 generalization did not change significantly when N1 was reclassified as wake. However, when N1 was reclassified as N2, generalization significantly improved from strong (0.82 ± 0.01) to excellent (0.87 ± 0.01) (*p* < 0.0001) with large effect size (*g* = 0.88). N3 remained strong for all groups, and *F* measures did not significantly differ. REM generalization remained strong across all groups, and marginally decreased when N1 was reclassified as wake, decreasing from 0.87 ± 0.01 to 0.86 ± 0.01 (*p* < 0.05) with miniscule effect size (*g* = 0.15). Overall, generalization benefited significantly for both versions of N1 reclassification. When reclassifying N1 as wake, overall generalization remained strong, significantly increasing from 0.85 ± 0.01 to 0.87 ± 0.01 (*p* < 0.0001) with medium effect size (*g* = 0.58). Reclassifying N1 as N2, on the other hand, significantly increased overall generalization to excellent (0.89 ± 0.01) (*p* < 0.0001) with large effect size (*g* = 1.2).

Simplifying human sleep stages to resemble those in rodent models had profound effects on generalization (Figure 4B). When N1 was reclassified as wake, and N2 and N3 were both reclassified as NREM (W1NREM23), wake generalization (0.86 ± 0.01) significantly increased in comparison with that of rodent data (0.95 ± 0.01, *p* < 0.0001) with large effect size (*g* = 1.27). Moreover, when N1, N2, and N3 were reclassified as NREM (NREM123), the *F* measure increase was still significant (0.92 ± 0.01, *p* < 0.0001) with medium effect size (*g* = 0.79). Interestingly, rodent data and W1NREM23 did not differ significantly in terms of NREM generalization. NREM123 reclassification, however, produced significantly higher generalization (0.95 ± 0.01) than rodent data (0.94 ± 0.01, *p* < 0.01) with medium effect size (*g* = 0.51). REM generalization was highest for rodent data (0.91 ± 0.01) and was significantly lower for both W1NREM23 (0.86 ± 0.01, *p* < 0.0001) and NREM123 (0.87 ± 0.01, *p* < 0.01) configurations, with large (*g* = 0.82) and medium (*g* = 0.59) effect sizes, respectively. Remarkably, overall generalization did not differ significantly between rodent data and NREM123 reclassification, resulting in remarkable *F* measures of 0.95 ± 0.01 and 0.94 ± 0.01, respectively. Generalization performance was marginally lower for W1NREM23 reclassification (0.91 ± 0.01, *p* < 0.001) with large effect size (*g* = 1.18). These results indicate that reclassification of N1 to either wake or N2, improved overall algorithm generalization. In comparison to rodent NREM classification, the results indicate that N1 is more similar to N2 and N3, than wake. Furthermore, performance with human and rodent data was similar despite different epoch sizes.

#### Impact of Training Set Size

**FIGURE 5:**
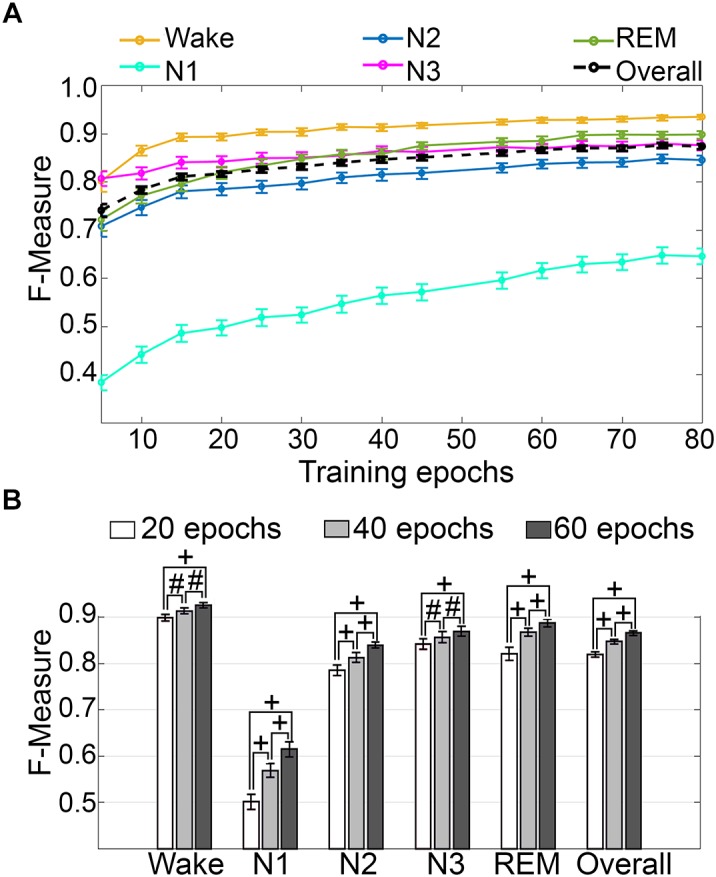
Impact of training set size on generalization of human recordings. **(A)** Training set size to *F*-measure response curves challenging set guidelines for human recordings (*n* = 52). Training epochs refers to training set size per vigilance state. **(B)** The impact of three different training set sizes on the generalization of all pooled human recordings. Data analyzed by paired t-test, and represented as mean ± SEM. #*p* < 0.01; +*p* < 0.001.

Training set size to F-measure response curves were generated for pooled human recordings, inclusive of transition epochs, which encompass an average of 1315 ± 24.21 total epochs per recording (*n* = 52) (Figure 5A). Generalization on most vigilance states peaked immediately in spite of moderate training set sizes, and started to plateau after ∼20 training epochs per stage, with the exception of N1, which steadily increased. Generalization for all pooled human recordings (*n* = 52) was compared in three different conditions: (i) 20 training epochs per stage, the minimum to reach overall strong generalization for most vigilance states; (ii) 40 epochs per stage, the guideline minimum for epoch lengths of 30 s; and (iii) 60 training epochs per stage, the maximum number assumed to still provide user-friendly training. Generalization increased dose-dependently with training set size, consistent with the theory of machine learning. However, benefits with larger training set sizes were modest, producing effect sizes from miniscule to medium (Figure 5B). Training set sizes as small as 20 epochs generated strong generalization (>0.80) on most stages, while at 40 epochs, all stages besides N1 generalized from strong to excellent (0.80–0.90).

#### Scoring Times

Computational times for automated wake-sleep stage scoring of recordings were 14.98 ± 0.07 s (UOH; *n* = 28; mean recording length 11.75 ± 0.17 h) and 14.95 ± 0.15 s (UMH; *n* = 24; mean recording length 10.04 ± 0.30 h).

### Validation of the Algorithm With Mouse and Rat Data

#### Generalization Across Experimental Groups

**FIGURE 6:**
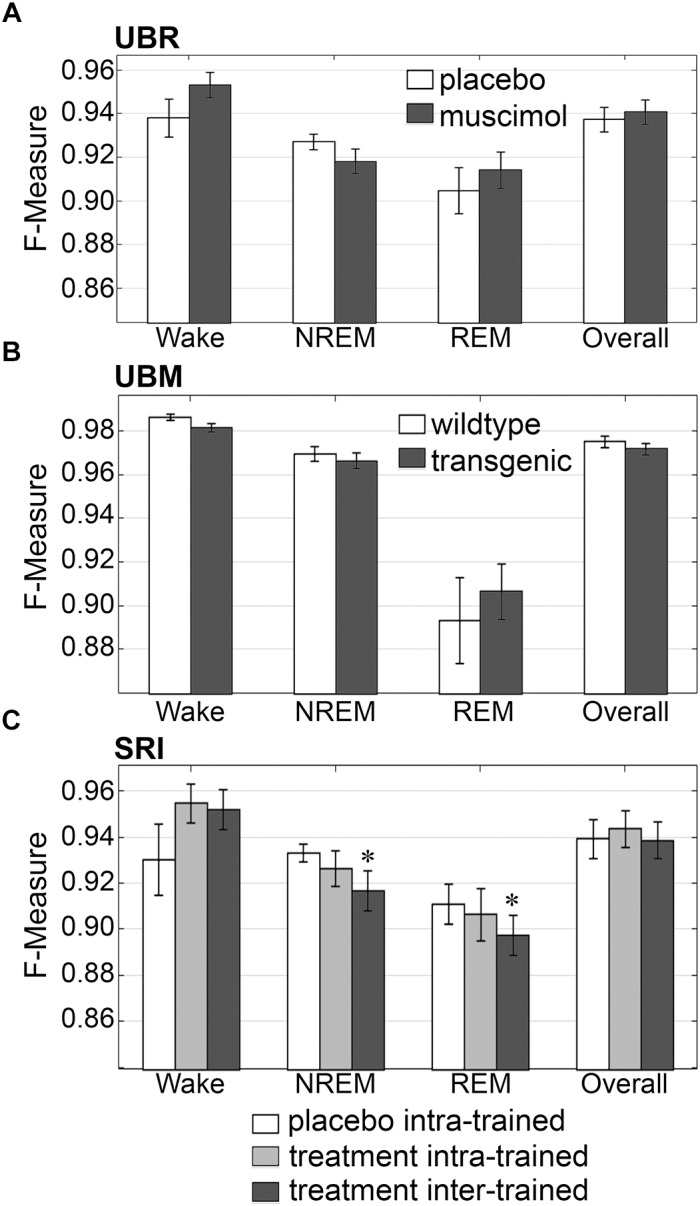
Generalization across rodent control and experimental UBR, UBM and SRI groups. Generalization (*F*-measure) of **(A)** UBR vehicle and muscimol treatment groups (*n* = 8); **(B)** UBM wildtype and hypocretin-ataxin3 transgenic narcoleptic mouse groups (*n* = 9); and **(C)** SRI pooled vehicle-treated and drug-treated groups (*n* = 11). Data analyzed by uncorrected paired *t*-test, and represented as mean ± SEM. ^∗^*p* < 0.05.

Firstly, the effects of treatment and genetic phenotype on generalization accuracy of rodent recordings were assessed. Across experimental groups of the UBR cohort, generalization of recordings from control rats, or those that received muscimol microinjection in the RVMM, did not significantly differ for any individual sleep stage or overall (*p* > 0.05) (Figure 6A), and all effect sizes were small to miniscule. On average, generalization scored ∼0.95 for wake, ∼0.93 for NREM, ∼0.91 for REM and ∼0.94 overall. For the UBM cohorts, generalization performance was compared between wildtype and transgenic mice. Similarly, *F*-measures did not significantly differ for any individual sleep stage or overall (Figure 6B) (*p* > 0.05), and all effect sizes were miniscule. On average, generalization was > 0.98 for wake, >0.96 for NREM, ∼0.90 for REM and >0.97 overall.

Similarly, generalization did not significantly differ for any individual sleep stage or overall for the SRI cohorts that received caffeine, zolpidem, or almorexant treatment, if Bonferroni correction was applied. With no correction however, small, but significant decreases in generalization were observed for NREM (0.93 ± 0.01 to 0.92 ± 0.01; *p* < 0.05; medium effect size [*g* = 0.72]) and REM sleep (0.91 ± 0.01 to 0.90 ± 0.01; *p* < 0.05; small effect size [*g* = 0.46]), when baseline only trained training sets were used (Figure 6C). Notably, *F*-measure for wake displayed a positive, non-significant trend for both treatment groups compared to vehicle (*p* = 0.15 and 0.14; medium effect sizes *g* = 0.58 and 0.51, respectively), when longitudinal- and baseline only trained training sets were used. Under no circumstance did longitudinal- versus baseline only trained treatment groups differ significantly (*p* > 0.05; with effect sizes above miniscule). Overall generalization across groups was ∼0.95 for wake, ∼0.93 for NREM, ∼0.91 for REM and ∼0.94 overall. These results indicate that experimental protocols, such as medullary inactivation, transgenic ablation of hypocretinergic neurons resulting in narcolepsy, or pharmacological treatment, have negligible effects on algorithm generalization accuracy of wake-sleep stage scoring of rodent data.

#### Impact of Transition Epochs

**FIGURE 7:**
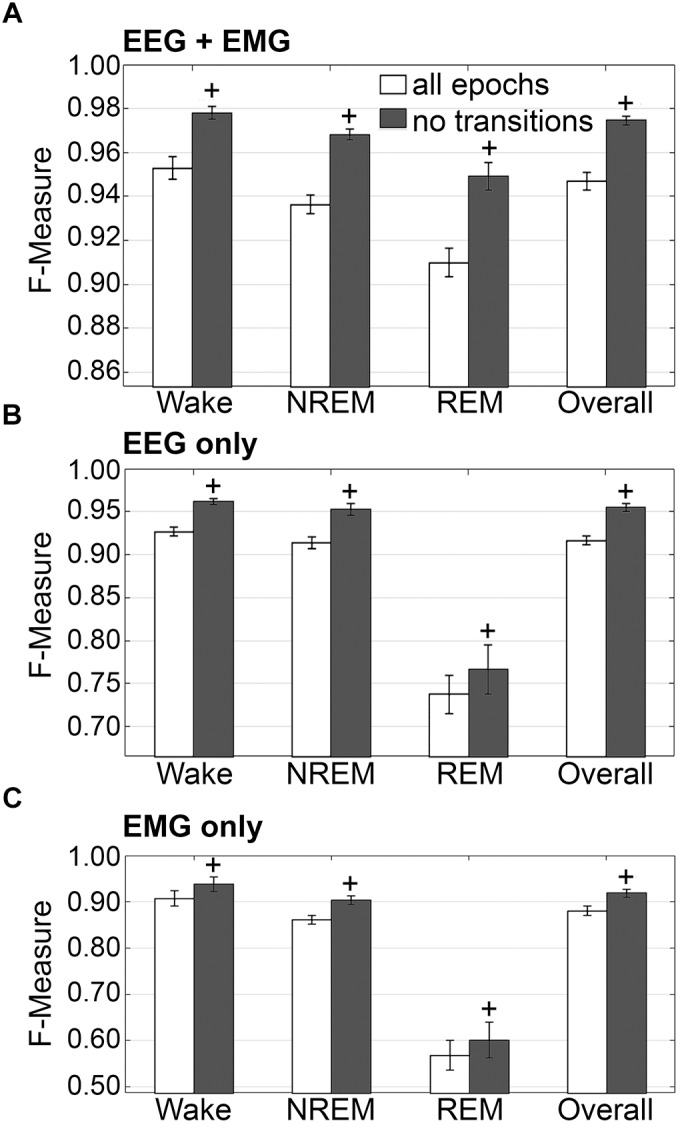
Impact of transition epochs on generalization of rodent recordings. Generalization of all rodent recordings (*n* = 56) trained with **(A)** both EEG and EMG; **(B)** EEG only and **(C)** EMG only, with and without consideration of transition epochs. Data analyzed by paired *t*-test compared to training with all epochs, and represented as mean ± SEM. ^+^*p* < 0.001.

The impact of transition epochs on generalization was also assessed on pooled rodent recordings (*n* = 54). Automated removal of transition epochs resulted in exclusion of 13.0 ± 0.89% of total epochs, which had a positive impact on the already high level generalization (Figure 7A). Wake *F*-measure significantly increased from 0.95 ± 0.01 to 0.98 ± 0.01 (*p* < 0.0001) with a large effect size (*g* = 0.82). NREM F-measure significantly increased from 0.94 ± 0.001 to 0.97 ± 0.01 (*p* < 0.0001) with a large effect size (*g* = 1.27). REM F-measure significantly increased from 0.91 ± 0.01 to 0.95 ± 0.01 (*p* < 0.0001) with a large effect size (*g* = 0.82); while overall *F*-measure significantly increased from 0.95 ± 0.01 to 0.97 ± 0.01 (*p* < 0.0001) with a large effect size (*g* = 1.16).

The benefits of removing transition epochs from the computation of *F*-measures on classifiers that relied on EEG only for training, were significant (Figure 7B). Wake *F*-measure significantly increased from 0.91 ± 0.02 to 0.94 ± 0.02 (*p* < 0.0001) with a small effect size (*g* = 0.26). NREM F-measure significantly increased from 0.86 ± 0.01 to 0.90 ± 0.01 (*p* < 0.0001) with medium effect size (*g* = 0.61). REM F-measure significantly increased from 0.57 ± 0.03 to 0.60 ± 0.04 (*p* < 0.001) with a minor effect size (*g* = 0.13); while the overall *F*-measure significantly increased from 0.88 ± 0.01 to 0.92 ± 0.01 (*p* < 0.0001) with a medium effect size (*g* = 0.54).

When comparing ‘all-epochs’ versus ‘no-transition epochs’ configurations with the standard configuration (EEG+EMG trained, all epochs considered), NREM EEG-only trained with no-transition epochs was the only metric that was not significantly different (with a minor effect size) and therefore returned to baseline generalization, in spite of lacking EMG data.

The benefits of excluding transition epochs from the computation of *F*-measures on classifiers that relied on EMG only for training, were significant (Figure 7C). Wake *F*-measure significantly increased from 0.91 ± 0.02 to 0.94 ± 0.02 (*p* < 0.0001) with a small effect size (*g* = 0.26). NREM *F*-measure significantly increased from 0.86 ± 0.01 to 0.90 ± 0.01 (*p* < 0.0001) with medium effect size (*g* = 0.61). REM *F*-measure significantly increased from 0.57 ± 0.03 to 0.60 ± 0.04 (*p* < 0.001) with a miniscule effect size (*g* = 0.13). Overall *F*-measure significantly increased from 0.88 ± 0.01 to 0.92 ± 0.01 (*p* < 0.0001) with a medium effect size (*g* = 0.54). Thus, as revealed in the human data analyses, automated ‘tagging’ of transition epochs (in addition to artifacts) for subsequent refinement of the machine learning process would potentially increase algorithmic accuracy for all stages and overall.

#### Generalization on Learning With the EEG or EMG Channel Only

**FIGURE 8:**
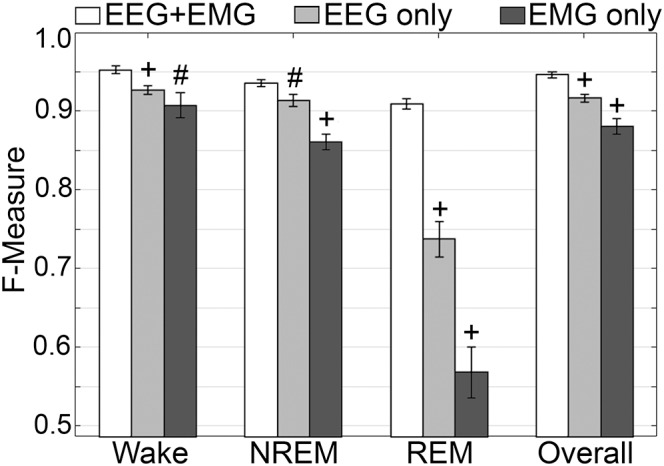
Impact of different training channel configurations on generalization of rodent recordings. Generalization of all rodent recordings (*n* = 56) across three different input channel configurations: EEG+EMG; EEG only; EMG only. Data analyzed by paired *t*-test compared to EEG+EMG, and represented as mean ± SEM. #*p* < 0.01; +*p* < 0.001.

Generalization was examined for pooled rodent recordings (*n* = 54), inclusive of transition epochs, under different conditions of input channel training (EEG+EMG [A]; EEG alone [B]; EMG alone [C]) (Figure 8). Different input channel configurations had a significant, albeit moderate, impact on wake *F*-measure. Generalization of wake stages significantly decreased from 0.95 ± 0.01 [A] to 0.93 ± 0.01 [B] (*p <* 0.0001), and 0.91 ± 0.02 [C] (P ≤ 0.001), with medium effect sizes (*g* = 0.68 and 0.51, respectively). The impact on NREM was more pronounced, where F-measure significantly decreased from 0.94 ± 0.01 [A] to 0.91 ± 0.01 [B] (P ≤ 0.001), and 0.86 ± 0.01 [C] (*p <* 0.0001), with medium and large effect sizes (*g* = 0.51 and 1.34, respectively). As expected, REM was most affected, where *F*-measure significantly decreased from 0.91 ± 0.01 [A] to 0.74 ± 0.02 [B] (*p <* 0.0001), and 0.57 ± 0.03 [C] (*p <* 0.0001), with medium and large effect sizes (*g* = 1.4 and 1.98, respectively). Overall, the decrease in generalization was minimal, with *F*-measure significantly decreased from 0.95 ± 0.01 [A] to 0.92 ± 0.01 [B] (*p <* 0.0001), and 0.88 ± 0.01 [C] (*p <* 0.0001), with large effect sizes (*g* = 0.86 and 1.18, respectively).

#### Impact of Training Set Size

**FIGURE 9:**
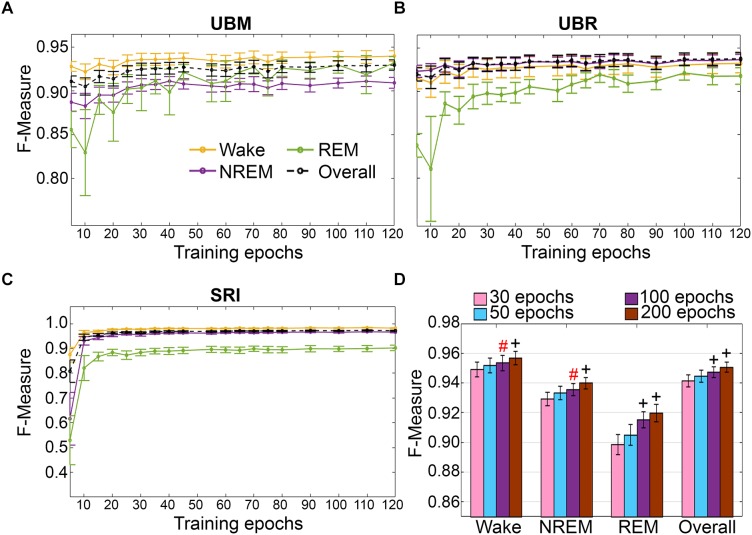
Impact of training set size on the generalization of rodent cohorts. Training set size to *F*-measure response curves challenging set guidelines for **(A)** UBM (*n* = 18), **(B)** UBR (*n* = 16), and **(C)** SRI cohorts (*n* = 22). Training epochs refers to training set size per each vigilance state. **(D)** The impact of four different training set sizes on the generalization of pooled rodent recordings (*n* = 56). Data analyzed by paired *t*-test compared to 30 epochs, and represented as mean ± SEM. ^#^*p* < 0.01; ^+^*p* < 0.001. Symbol color represents effect sizes, red, miniscule; black, small.

Training set size to *F*-measure response curves were generated for each rodent cohort, inclusive of transition epochs, which comprise 27000 (UBM; 4 s epochs), 21600 (UBR; 4 s epochs) and 2954 ± 226.7 (SRI; 10 s epochs) total epochs per recording. A similar trend was observed across UBM, UBR and SRI cohorts (Figure 9A–C). Generalization on most vigilance states peaked immediately, in spite of very moderate training set sizes, and started to plateau after ∼30–50 training epochs. Generalization for pooled rodent recordings (*n* = 54) was compared between four different conditions: (i) 30 training epochs, the minimum to reach *F*-measure ≥ 0.90 for all vigilance states; (ii) 50 epochs, the guideline minimum for epoch lengths of 10 s (SRI cohort); (iii) 100 epochs, the guideline minimum for epoch lengths of 4 s (UBM and UBR cohorts); and (iv) 200 training epochs, the maximum assumed to still provide user-friendly training. Generalization increased dose-dependently with training set size, consistent with the theory of machine learning. However, benefits with larger training set sizes were modest (Figure 9D).

#### Scoring Times

Computational times for automated wake-sleep stage scoring of recordings were 7.07 ± 0.05 s (UBM; *n* = 18; 30 h recordings), 6.04 ± 0.06 s (UBR; *n* = 16; 24 h recordings) and 2.04 ± 0.02 s (SRI; *n* = 22; mean recording length 8.81 ± 0.63 h).

### Validation of the Algorithm With Pigeon Data

Because of the lengthy duration and difficult nature of scoring avian sleep (Lesku et al., 2009), data from only 5 pigeons with prior manual scoring were used for validation. Despite the small dataset, algorithm generalization from EEG and ACC inputs was excellent overall and exhibited high agreement with manual scoring for wakefulness and NREM. Performance of the algorithm for identifying REM was somewhat lower, which would necessitate a follow-up inspection of video recordings during short EEG activations to check whether the eyes were closed, along with behavioral signs of reduced skeletal muscle tone that are more likely to reflect REM than wakefulness.

#### Impact of Transition Epochs

**FIGURE 10:**
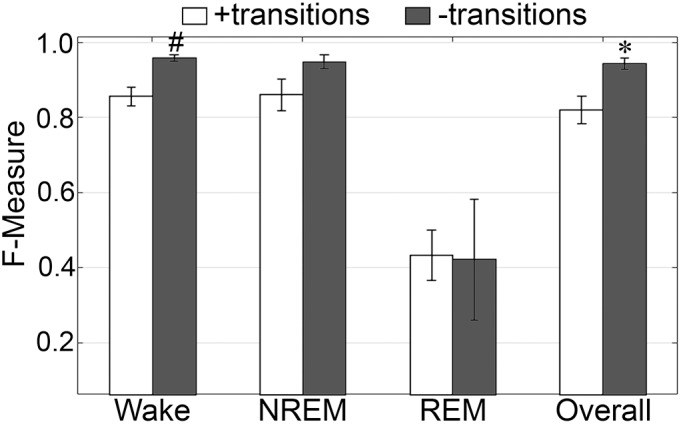
Impact of transition epochs on generalization of pigeon recordings. Generalization of all pigeon recordings (*n* = 5), with and without consideration of transition epochs. Data analyzed by paired t-test compared to training with all epochs, and represented as mean ± SEM. ^∗^*p* < 0.05; ^#^*p* < 0.01.

The impact of automated exclusion of transition epochs on generalization was assessed (Figure 10). Automated removal of transition epochs resulted in 20.5 ± 1.44% of total epochs excluded. Wake *F*-measure significantly increased from 0.96 ± 0.006 to 0.99 ± 0.002 (*p <* 0.01) with a large effect size (*g* = 2.33). NREM F-measure also marginally increased from 0.97 ± 0.01 to 0.99 ± 0.005, although this was not significantly different (*p* = 0.09). REM F-measure was unchanged, 0.86 ± 0.02 to 0.86 ± 0.04 (*p* = 0.95); while overall generalization significantly increased from 0.96 ± 0.009 to 0.99 ± 0.004 (*p <* 0.05) with a large effect size (*g* = 1.79). In practical terms, refinement of the machine learning process using transition epochs would result in marginally increased accuracy for all stages, except REM.

#### Generalization on Training With EEG or Accelerometer Channels Only

**FIGURE 11:**
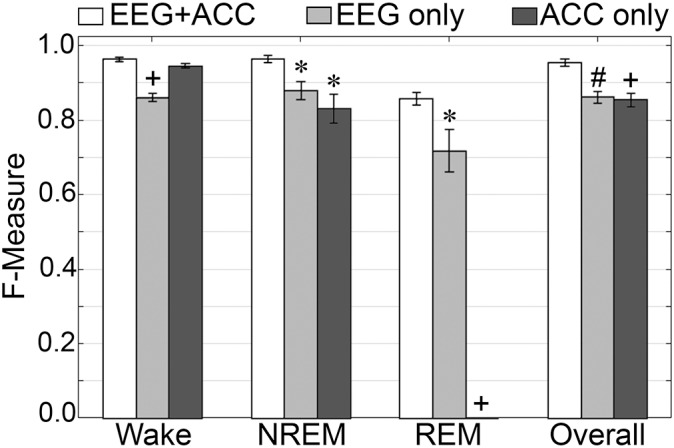
Impact of different training channel configurations on generalization of pigeon recordings. Generalization of all pigeon recordings (*n* = 5) across three different input channel configurations: EEG+ACC; EEG only; ACC only. Data analyzed by paired *t*-test compared to EEG+EMG, and represented as mean ± SEM. ^∗^*p* < 0.05; ^#^*p* < 0.01.

*F*-measure generalization was computed for all pigeon recordings (*n* = 5) under different conditions of input channel training (EEG+ACC [A]; EEG-alone [B]; ACC-alone [C]) (Figure 11). EEG-alone had a significant impact on wake classification, whereas ACC-alone reliably classified this stage. Wake F-measures significantly decreased from 0.96 ± 0.006 [A] to 0.86 ± 0.01 [B] (*p <* 0.0001), and 0.95 ± 0.006 [C], which was not significantly different (*p* = 0.07), with large effect sizes (*g* = 4.42 and 1.19, respectively). The impact on NREM was significant for both configurations, where *F*-measure decreased from 0.97 ± 0.01 [A] to 0.88 ± 0.02 [B] (*p <* 0.05), and 0.83 ± 0.04 [C] (*p <* 0.05), with large effect sizes (*g* = 1.80 and 1.86, respectively). REM was also affected by EEG alone and could not be classified on ACC alone, where *F*-measure significantly decreased from 0.86 ± 0.02 [A] to 0.71 ± 0.06 [B] (*p <* 0.05), with large effect size (*g* = 1.34). Overall, the decrease in generalization was, in fact, similar with both configurations, with F-measure significantly decreased from 0.96 ± 0.009 [A] to 0.86 ± 0.02 [B] (*p <* 0.01), and 0.86 ± 0.02 [C] (*p <* 0.0001), with large effect sizes (*g* = 2.87 and 2.89, respectively).

#### Impact of Training Set Size

**FIGURE 12:**
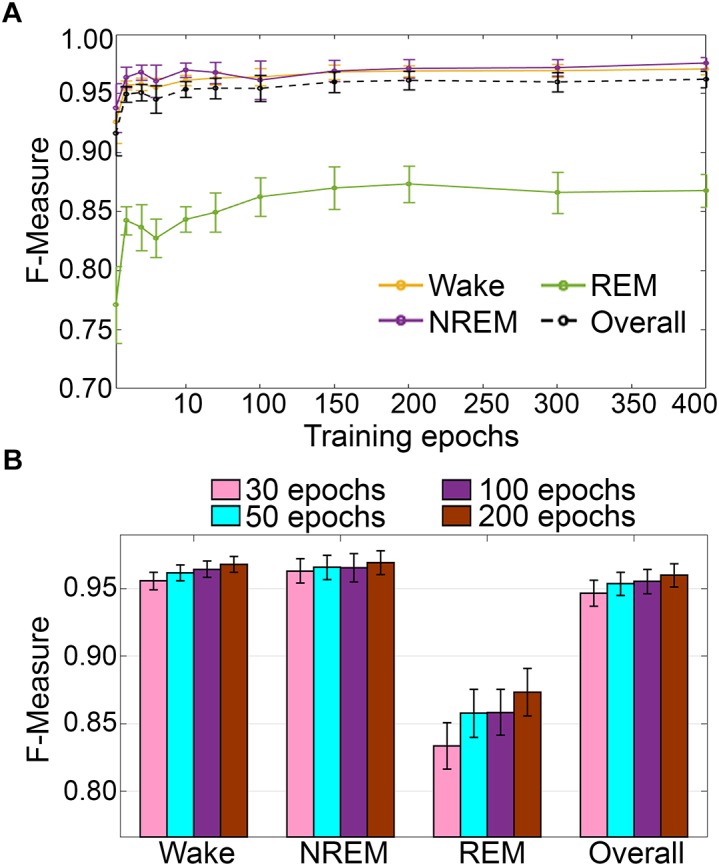
**(A)** Impact of training set size on the generalization of pigeon recordings. Training set size to *F*-measure response curves challenging set guidelines for pigeon recordings (*n* = 5). Training epochs refers to training set size for each vigilance state. **(B)** There were no significant effects of four different training set sizes on the generalization of pooled pigeon recordings. Data analyzed by paired *t*-test compared to 30 epochs, and represented as mean ± SEM.

Training set size to *F*-measure response curves were generated for the pigeon recordings (*n* = 5) using the standard configuration inclusive of transition epochs, which comprised an average of 45105 ± 7270 total epochs (4 s) per recording (Figure 12A). Generalization on most vigilance states peaked immediately, and started to plateau after ∼20–40 training epochs, with the exception of REM, which steadily increased in spite of moderate training set sizes. Similar to the rodent data validation, generalization for pigeon recordings was compared under four different conditions: 30, 50, 100, and 200 training epochs (Figure 12B). Generalization F-measure performance increased size-dependently with training set size. However, benefits with larger training set sizes were modest for most stages, whereas REM generalization peaked at 200 epochs (Figure 12B).

#### Scoring Times

Computational time for automated wake-sleep stage scoring of recordings was 15.71 ± 5.65 s (*n* = 5; mean recording length 50.11 ± 8.1 h).

## Discussion

Somnivore was developed as a multi-layered system capable of learning from limited training sets, using large input space dimensionalities from a rich variety of polysomnography inputs, to provide automated wake-sleep stage scoring with rapid computational times. As one of the goals for Somnivore was fast processing speed, design of an *a priori* selected feature set required a machine learning algorithm capable of integrating a large feature space. Other algorithms validated so far have been limited by use of small group sizes for validation (Crisler et al., 2008; Sinha, 2008; Gross et al., 2009; McShane et al., 2013; Sunagawa et al., 2013), analyzed recordings using non-standard recording conditions (Koley and Dey, 2012; Stepnowsky et al., 2013; Kaplan et al., 2014; Wang et al., 2015), or were validated using baseline healthy or control subjects only (Stephenson et al., 2009; Khalighi et al., 2012; Koupparis et al., 2014; Kreuzer et al., 2015; Rempe et al., 2015; Gao et al., 2016), which has restricted their wider implementation in sleep research.

Automated sleep scoring has a rich, but varied history. Multiple algorithms have been proposed, tested and validated across a limited, but variable number of conditions. Several have produced remarkable results in terms of generalization (Crisler et al., 2008; Sunagawa et al., 2013; Bastianini et al., 2014), which poses the question of why these procedures have not become established in the sleep research field. Thus, we developed Somnivore to address many of the perceived shortcomings of earlier systems. One of the major criticisms of previous systems that attempted to automatically score sleep states was that they were only validated on baseline, wildtype or control (placebo/vehicle) data. Subject treatments often alter signal features that many such algorithms rely on, which leads to inaccurate generalization. However, it is rare for sleep scientists to only examine baseline, wildtype or control data. The current study is the first of its kind to validate an algorithm for automated wake-sleep stage scoring using large and diverse datasets collected from multiple species, using diverse methods and differing epoch sizes, under different treatment or genetic conditions. All recordings were used for the validation studies and no outliers were removed. The subjects were sourced from multiple, independent laboratories, in which they were manually scored by members of these laboratories, with some subject data extracted from published studies. This study design reduces perceived bias or conflict of interest.

In the rodent data analyzed, *F*-measure-based generalization was high and unchanged by pharmacological treatment or genetically induced sleep impairment (narcolepsy). Moreover, the absolute *F*-measure generalization was consistently > 0.90 across all wake-sleep states and overall, which was further increased by exclusion of transition epochs.

In the human data analyzed, results were comparable with inter-scorer agreement, with minor discrepancies. For the UMH recordings, generalization was unchanged between control and alcohol conditions, although a minor decrease was detected for the baseline only trained alcohol condition. Nonetheless, generalization remained strong to excellent across all stages (except for N1) and overall. In UOH recordings, generalization was analogous between HOA and HYA, but MOA recordings registered lower generalization, particularly for N1 and N3. Nonetheless, generalization for MOA recordings also remained average to excellent for all stages (except for N1). Reclassification of N1 to either wake or N2, or simplification to resemble rodent wake-sleep stages, revealed that the N1 stage is more similar to N2 and N3 than wake. Furthermore, automated scoring of human and rodent data were similar despite different epoch sizes. Similarly, analyses of human and rodent data revealed that variability of algorithm-generated automated wake-sleep stage scoring largely lies in the determination of transition epochs. Thus, in practical terms, it would be worthwhile to include an additional analysis process following the initial automated scoring process, whereby the machine learning process is further refined by manual correction of transition epochs and artifacts by automated tagging of these epochs, which would provide substantial increases in the final automated scoring accuracy. Because of the nominal training set sizes needed to achieve strong generalization, and the rapid automated scoring process of Somnivore (∼15 s for 10–12 h human recordings, ∼7 s for 30 h rodent recordings, and ∼16 s for ∼50 h pigeon recordings), manual assessment and correction of tagged transition and artifact epochs would be minimal additional work flow, relative to manual scoring.

While rodent and human data constitutes the overwhelming bulk of polysomnography sleep data collected for research, studies are also conducted in less conventional animal models, such as dogs (Pickworth et al., 1982), cats (Lancel et al., 1991), pigeons (Tobler and Borbely, 1988), penguins (Buchet et al., 1986), dolphins (Oleksenko et al., 1992), seals (Mukhametov et al., 1985), and non-human primates (Crowley et al., 1972). Therefore, for comprehensive validation of the flexibility of Somnivore, a diverse array of recordings was tested, including data from experimental pigeons. In this regard, the timescale of REM episodes in birds is markedly different from that observed in most mammals. In mammals, REM bouts are typically minutes or tens of minutes long, whereas in birds, REM episodes are rarely longer than 16 s (Lesku and Rattenborg, 2014). The validation of Somnivore on such diverse species indicates that the algorithm is flexible and sensitive enough to autoscore typical and atypical polysomnographic sleep data.

Accurate sleep scoring relies on the quality of polysomnographic data. Unfortunately, due to the nature of signal acquisition, signals often become corrupted and unusable. Manual scoring offsets this issue using the excellent pattern recognition abilities of humans; and ultimately this is a major reason why this procedure has remained the gold standard. All automated scoring algorithms validated thus far have analyzed high quality polysomnography data, and none have specifically reported using compromised input data. Thus, it was of interest to assess the capability of Somnivore to score polysomnography data on limited inputs. Across all species, performance decreased considerably for REM sleep when the EEG or EMG was removed from the learning model, as expected, though remarkably, overall *F*-measures remained > 0.90 and ∼0.87 for EEG-only and EMG-only trained classification, respectively. Overall, generalization was increased through automated exclusion of transition epochs (the first and last epoch of each sleep stage bout). When transition epochs were excluded from EEG-only trained classification, the detrimental effects of removing the EMG were normalized in the case of NREM, and recovered for REM, albeit modestly. The same was observed for EMG-only classification, although REM scoring became unreliable (*F*-measure ∼0.60). Overall, this indicates that in the absence of EMG data, Somnivore is still able to provide adequate generalization, while in the case of missing EEG data, adequate generalization can only be maintained for wake and NREM. The case for which the exclusion of transition epochs produced the most tangible benefit was the standard EEG+EMG classification. In spite of generalization being already excellent (wake = 0.95; NREM = 0.94; REM = 0.91; overall = 0.95), exclusion of transition epochs raised generalization to unprecedented F-measures of 0.98, 0.97, and 0.95 for wake, NREM and REM, respectively, leading to an overall generalization of 0.97. This outcome was achieved with scoring times ranging from ∼2 s (SRI) to 7 s (UBM) of a large sample size (*n* = 54).

Somnivore’s development was pursued with the general aim of providing reliable generalization toward the current gold-standard of manual, visual scoring, while preserving user-friendliness. Appropriate metrics of user-friendliness in a supervised machine learning based automated sleep scoring protocol pertain to technical settings and the amount of manual scoring of training sets required. Somnivore’s technical settings were designed to be minimal, and it mainly relies on consistency scoring rules, the channels used for training, and chronotype settings, which were all kept as default configurations. Analysis of training-set sizes relative to generalization response curves, indicated that a minimal amount of training was required to produce adequate generalization for all species. Thus, training-set sizes of as little as 30 epochs per stage for rodent data produced *F*-measure generalization > 0.90 on all wake-sleep stages and overall. This equated to 2 min (4 s epochs) to 5 min (10 s epochs) of training per stage. For human data, 20 epochs (i.e., 10 min) of training per stage produced strong generalization across all wake–sleep stages (except for N1) and overall.

### Limitations

Comprehensive inter-scorer agreement analysis was conducted on human data, showcasing how inter-scorer agreement between manual hypnograms and their associated automatically scored hypnograms generated by Somnivore was comparable to the gold-standard inter-scorer agreement between two trained experts in the same laboratory. Our findings highlighted inherent problems within the scoring of human stage N1. However, inter-scorer agreement validation studies also confirmed previous literature reports that N1 is a volatile stage that systematically produces inadequate agreement even between trained experts, both within or outside the same laboratory (Wendt et al., 2015; Younes et al., 2016). Accordingly, Somnivore performed as well on N1 as reported in the literature for manually scored data (Younes et al., 2016). In this regard, inter-scorer agreement between MS1 and MS2 scorers for UMH data was inadequate (<0.50), resulting in variable algorithm learning and thus, variable algorithm generalization (Figure 2). Due to the high-throughput nature of Somnivore’s analyses of experimental end-measures, several novel, cautionary findings were extracted from the recordings provided by external laboratories for evaluation.

In regard to the analysis of avian sleep, the short timescale of REM episodes and maintenance of NREM-like muscle tone (Lesku and Rattenborg, 2014) may require each short episode of avian EEG activity to be checked against video recordings to distinguish REM from brief awakenings from sleep. Unlike wakefulness, REM is associated with eye closure and behavioral signs of reduced skeletal muscle tone, including head drops and swaying, that do not consistently manifest reduced muscle tone. This is a time-consuming process that has led some to sample the EEG to avoid the demands of continuous scoring (Lesku et al., 2011). Somnivore performed well at identifying REM, although its performance was lower compared to the excellent generalization of wake and NREM stages. Thus, it may be worthwhile to review the automated scoring for any necessary manual adjustments to short EEG activations that may (or may not) reflect REM.

## Conclusion

We developed a supervised machine learning algorithm, named Somnivore. Validation of the accuracy of algorithm generalization was evaluated using F-measure, which considers both precision and sensitivity (Powers, 2011). Somnivore generated excellent overall generalization across human, rodent and pigeon polysomnography sleep recordings. These included data from humans of both genders, younger and older subjects, young subjects after alcohol consumption, and older subjects with mild cognitive impairment; from standard experimental rats and mice (wildtype and transgenic hypocretin neuron-ablated), and recordings examining the effects of various pharmacological interventions, such as alcohol, muscimol-inactivation of medulla, caffeine, zolpidem, almorexant and placebo/vehicle; and from pigeons.

Somnivore’s generalization was also evaluated under conditions of ‘signal-challenged’ data, and provided excellent performance in all conditions using only one EEG channel for training/learning. Excellent generalization was observed with learning using only one EMG channel or two EOG channels for human recordings. Furthermore, validation studies highlighted that a substantial part of the disagreement between manual and automated hypnograms was associated with transition epochs. In this regard, Somnivore was designed to provide automated detection and exclusion of these epochs from analysis, which provides further control over automated wake-sleep stage scoring.

Somnivore, has been comprehensively validated as an accurate, high-throughput platform for automated classification of wake-sleep stages from diverse polysomnography data. Importantly, the flexibility of the analysis enables its use in a range of experimental situations, including studies of normal sleep to those for drug discovery, genetically modified rodent models, and sleep health in ecological studies. This analysis tool will enable faster insights for the improved treatment of primary sleep disorders and those associated with psychiatric and neurodegenerative diseases.

## Data Availability

The datasets generated for this study are available on request to the corresponding author.

## Author Contributions

GA and AG conceived the project. GA developed the machine learning algorithm, designed and performed the validation experiments, analyzed the data, and wrote the paper. SMa wrote the paper. DM, MC, FDV, SB, GZ, RA, and SMo performed, analyzed, and provided rodent data for validation studies. AA, SB, JL, NR, and AV performed, analyzed and provided pigeon data for validation studies. EW, KP, KW, RF, JC, and CN performed, analyzed and provided human data for validation studies. DF, LJ, and AG supervised the project, and wrote the paper.

## Conflict of Interest Statement

GA, SM, and AG are commercializing the Somnivore software. The remaining authors declare that the research was conducted in the absence of any commercial or financial relationships that could be construed as a potential conflict of interest.

## Footnotes

1 http://www.evolocus.com
