# Supplementary material for: Validation of ‘Somnivore’, a Machine Learning Algorithm for Automated Scoring and Analysis of Polysomnography Data
Source: Front Neurosci. 2019 Mar 18;13:207. doi: 10.3389/fnins.2019.00207 (PMC6431640; doi:10.3389/fnins.2019.00207)
Supplement: Supplementary file 1 [file Table_1.DOCX]

# 1. Supplementary Material

## 1.1 Table 1. Participant details of UOH cohort

| Subject | Status | Montage | Scorer | Age | Sex |
| --- | --- | --- | --- | --- | --- |
| 1 | HOA | EOGL:A2, EOGR:A1, EMGL:A1, EMGR:A1, Fz:A1, Cz:A1, Pz:A1, Oz:A1 | EJM | 69 | F |
| 2 | HOA | EOGL:A2, EOGR:A1, EMGL:A1, EMGR:A1, Fz:A1, Cz:A1, Pz:A1, Oz:A1 | EJM | 74 | M |
| 3 | HOA | EOGL:A2, EOGR:A1, EMGL:A1, EMGR:A1, Fz:A1, Cz:A1, Pz:A1, Oz:A1 | EJM | 71 | M |
| 4 | HOA | EOGL:A2, EOGR:A1, EMGL:A1, EMGR:A1, Fz:A1, Cz:A1, Pz:A1, Oz:A1 | EJM | 70 | M |
| 5 | HOA | EOGL:A2, EOGR:A1, EMGL:A1, EMGR:A1, Fz:A1, Cz:A1, Pz:A1, Oz:A1 | EJM | 66 | M |
| 6 | HOA | EOGL:A2, EOGR:A1, EMGL:A1, EMGR:A1, Fz:A1, Cz:A1, Pz:A1, Oz:A1 | EJM | 65 | F |
| 7 | HOA | EOGL:A2, EOGR:A1, EMGL:A1, EMGR:A1, Fz:A1, Cz:A1, Pz:A1, Oz:A1 | EJM | 73 | F |
| 8 | HOA | EOGL:A2, EOGR:A1, EMGL:A1, EMGR:A1, Fz:A1, Cz:A1, Pz:A1, Oz:A1 | EJM | 78 | M |
| 9 | HOA | EOGL:A2, EOGR:A1, EMGL:A1, EMGR:A1, Fz:A1, Cz:A1, Pz:A1, Oz:A1 | EJM | 72 | F |
| 10 | MOA | EOGL:A2, EOGR:A1, EMGL:A1, EMGR:A1, Fz:A1, Cz:A1, Pz:A1, Oz:A1 | EJM | 77 | M |
| 11 | MOA | EOGL:A2, EOGR:A1, EMGL:A1, EMGR:A1, Fz:A1, Cz:A1, Pz:A1, Oz:A1 | EJM | 76 | M |
| 12 | MOA | EOGL:A2, EOGR:A1, EMGL:A1, EMGR:A1, Fz:A1, Cz:A1, Pz:A1, Oz:A1 | EJM | 74 | M |
| 13 | MOA | EOGL:A2, EOGR:A1, EMGL:A1, EMGR:A1, Fz:A1, Cz:A1, Pz:A1, Oz:A1 | EJM | 81 | F |
| 14 | MOA | EOGL:A2, EOGR:A1, EMGL:A1, EMGR:A1, Fz:A1, Cz:A1, Pz:A1, Oz:A1 | EJM | 67 | M |
| 15 | MOA | EOGL:A2, EOGR:A1, EMGL:A1, EMGR:A1, Fz:A1, Cz:A1, Pz:A1, Oz:A1 | EJM | 84 | F |
| 16 | MOA | EOGL:A2, EOGR:A1, EMGL:A1, EMGR:A1, Fz:A1, Cz:A1, Pz:A1, Oz:A1 | EJM | 76 | F |
| 17 | HYA | EOG1:A2, EOG2:A1, EMG1:chin, EMG2:chin, Oz:A2, Pz:A2, C3:A2, Fz:A2, C4:A1 | KP | 20 | M |
| 18 | HYA | EOG1:A2, EOG2:A1, EMG1:chin, EMG2:chin, Oz:A2, Pz:A2, C3:A2, Fz:A2, C4:A1 | KP | 22 | F |
| 19 | HYA | EOG1:A2, EOG2:A1, EMG1:chin, EMG2:chin, Oz:A2, Pz:A2, C3:A2, Fz:A2, C4:A1 | KP | 22 | F |
| 20 | HYA | EOG1:A2, EOG2:A1, EMG1:chin, Oz:A2, Pz:A2, C3:A2, Fz:A2, C4:A1 | KP | 20 | M |
| 21 | HYA | EOG1:A2, EOG2:A1, EMG2:chin, Oz:A2, Pz:A2, C3:A2, Fz:A2, C4:A1 | KP | 21 | M |
| 22 | HYA | EOG1:A2, EOG2:A1, EMG1:chin, EMG2:chin, Oz:A2, Pz:A2, C3:A2, Fz:A2, C4:A1 | KP | 34 | F |
| 23 | HYA | EOG1:A2, EOG2:A1, EMG2:chin, Oz:A2, Pz:A2, C3:A2, Fz:A2, C4:A1 | KP | 24 | M |
| 24 | HYA | EOG1:A2, EOG2:A1, EMG2:chin, Oz:A2, Pz:A2, C3:A2, Fz:A2, C4:A1 | KP | n/a | M |
| 25 | HYA | EOG1:A2, EOG2:A1, EMG2:chin, Oz:A2, Pz:A2, C3:A2, Fz:A2, C4:A1 | KP | 25 | M |
| 26 | HYA | EOG1:A2, EOG2:A1, EMG2:chin, Oz:A2, Pz:A2, C3:A2, Fz:A2, C4:A1 | KP | 21 | M |
| 27 | HYA | EOG1:A2, EOG2:A1, EMG2:chin, Oz:A2, Pz:A2, C3:A2, Fz:A2, C4:A1 | KP | 24 | M |
| 28 | HYA | EOG1:A2, EOG2:A1, EMG2:chin, Oz:A2, Pz:A2, C3:A2, Fz:A2, C4:A1 | KP | 22 | F |

HOA = healthy older adult; MOA = mild cognitively-impaired older adult; HYA = healthy younger adults; n/a = not available
