# Supplementary material for: Validation of ‘Somnivore’, a Machine Learning Algorithm for Automated Scoring and Analysis of Polysomnography Data
Source: Front Neurosci. 2019 Mar 18;13:207. doi: 10.3389/fnins.2019.00207 (PMC6431640; doi:10.3389/fnins.2019.00207)
Supplement: Supplementary file 2 [file Table_2.DOCX]

# 1. Supplementary Material

## 1.2 Table 2. Summary of cohorts in rodent validation studies

| **Cohort** | **Recording 1** | **Recording 2** | **D** | **Species** | **Strain** | **Phenotype** | **n** |
| --- | --- | --- | --- | --- | --- | --- | --- |
| UBM-WT | Baseline | n/a | 30 | Mouse | C57Bl/6J | WT | 9 |
| UBM-TG | Baseline | n/a | 30 | Mouse | C57Bl/6J | TG | 9 |
| UBR-VEH | Vehicle × 6 | n/a | 24 | Rat | SD | WT | 8 |
| UBR-MU | Muscimol 1 mM × 6 | n/a | 24 | Rat | SD | WT | 8 |
| SRI-CAF | Vehicle | Caffeine 10 mg/kg | 6 | Rat | SD | WT | 7 |
| SRI-ZOL | Vehicle | Zolpidem 30 mg/kg | 12 | Rat | SD | WT | 2 |
| SRI-ALM | Vehicle | Almorexant 100 mg/kg | 12 | Rat | SD | WT | 2 |

D = duration recording (h); SD = Sprague-Dawley; TG *=* transgenic; WT = wild type
